# Integrated Analysis of FAM57A Expression and Its Potential Roles in Hepatocellular Carcinoma

**DOI:** 10.3389/fonc.2021.719973

**Published:** 2021-11-01

**Authors:** Junwei Wei, Yun Liu, Caiyan Zhao

**Affiliations:** ^1^ Department of Infectious Diseases, The Third Affiliated Hospital of Hebei Medical University, Shijiazhuang, China; ^2^ Department of Gastroenterology, The First Hospital of Handan City, Handan, China; ^3^ Department of General Surgery, The First Hospital of Handan City, Handan, China

**Keywords:** FAM57A, hepatocellular carcinoma, expression level, oncogene, functional assay

## Abstract

**Background:**

Family with sequence similarity 57 member A (FAM57A) is a membrane associated gene contributing to lung carcinogenesis. In hepatocellular carcinoma (HCC) and other cancers, whether FAM57A exerts similar roles has been rarely reported. Herein, the biological functions and clinical significance of FAM57A in HCC were explored.

**Methods:**

Initially the differential expression of FAM57A between nontumor and HCC tissues was validated using a number of publicly accessible databases and immunohistochemistry (IHC). Then, the Kruskal–Wallis rank sum test or the Wilcoxon rank sum test as well as logistic regression were employed to analyze the association of FAM57A expression with clinical characteristics of HCC. The Cox regression and Kaplan–Meier analyses were conducted to assess the prognostic significance. Besides, with the coexpression analysis, Gene Ontology (GO), Gene Set Enrichment Analysis (GSEA), and Kyoto Encyclopedia of Genes and Genomes (KEGG) enrichment analysis, the molecular pathomechanisms that were mediated by FAM57A in HCC were elucidated. Furthermore, the correlations between FAM57A expression and tumor-infiltrating immune cells (TIICs) or immune checkpoint genes were analyzed. Finally, *in vitro* cell functional assay was carried out to preliminarily verify the role of FAM57A in HCC.

**Results:**

FAM57A expression was demonstrated to be higher in HCC samples than in nontumor samples (all *p*-values <0.05), statistically correlated with clinicopathological characteristics (clinical stage, T stage, pathological grade), and inversely correlated to HCC patient survival. Univariate and multivariate Cox regression analyses showed that FAM57A expression could independently predict prognosis in HCC patients. Functional enrichment analyses further indicated that FAM57A was involved in multiple tumor-related pathways. FAM57A expression was positively correlated with TIICs, gene markers of TIICs, as well as immune checkpoint genes. Also, high expression of FAM57A predicted a poor prognosis for HCC based on immune cell subgroups. Functional assay of FAM57A knockdown significantly inhibited cell proliferation and induced cell apoptosis in HCC cells.

**Conclusions:**

Our results indicated that FAM57A could be used as a biomarker to predict the prognosis and immunotherapy response for HCC patients and might function as an oncogene to promote HCC progression.

## Introduction

Hepatocellular carcinoma (HCC) is the sixth most frequently diagnosed cancer and the fourth leading cause of cancer-related deaths worldwide (1). While treatment strategies for liver cancer have expanded with the emergence of new therapies, the prognosis of patients with advanced stage disease is typically measured in months (2). Even in the fraction of patients eligible for surgical treatment, less than 7.2% of HCC patients survived for 10 years (3). Therefore, identifying a biomarker that not only could accurately predict prognosis but also could be transformed into an HCC treatment option would be ideal. Bioinformatics analysis and analysis of gene expression have elucidated the pathomechanism of certain cancers, revealing biomarkers for predicting prognosis and potentiating drug discovery and development (4, 5).

The FAM57A gene modulates glutathione metabolism and the transport of amino acids (6). Previous studies have shown that FAM57A knockdown inhibited the proliferation of the human lung adenocarcinoma cells (SPCA1) *in vitro* and suppressed the tumorigenicity in the nude mice *in vivo* (7). Additionally, FAM57A has been demonstrated to activate the PI3K/Akt and Raf/Mek/Erk cascades and upregulate the expression of genes promoting metastasis in lung cancer cells (8). These results indicated that FAM57A contributes to lung tumorigenesis. There have also been reports that indicated FAM57A could play an oncogenic role in head and neck squamous cell cancer (9, 10). To date, the impact of FAM57A in HCC has rarely been reported. Furthermore, cancer immunotherapy has literally exploded in the understanding of how to engage the immune system to fight against cancer and the development of modern cancer immunotherapies, including HCC. Specifically, it is possible that tumor-infiltrating immune cells (TIICs) could affect the efficacy of immunotherapy. However, the relationship between FAM57A expression and immune infiltration in HCC remains unclear.

In the present study, the differential expression of FAM57A in HCC tissues and nontumor tissues was validated using several publicly accessible databases and immunohistochemistry (IHC) of our tissue samples. The clinical and prognostic value of FAM57A expression was then analyzed, and multiple enrichment analyses were performed to investigate the potential molecular mechanisms that could be mediated by FAM57A in HCC. Subsequently, we investigated the correlation between FAM57A expression and TIICs and immune checkpoint genes. Based on the results of the bioinformatics analysis and IHC, we determined that FAM57A could potentially serve as a biomarker to predict the prognosis of and immunotherapy response to HCC, and it might function as an oncogene in the progression of HCC. Therefore, we finally conducted a loss-functional experiment to preliminarily verify the role of FAM57A in proliferation of HCC cells.

## Materials And Methods

### FAM57A Expression Analysis

To explore the expression pattern of FAM57A in HCC, the Gene Expression Omnibus (GEO), the International Cancer Genome Consortium (ICGC), and The Cancer Genome Atlas (TCGA) datasets were utilized. We downloaded TCGA transcriptome, DNA methylation, and corresponding clinical data for HCC patients from the Genomic Data Commons Data Portal (https://portal.gdc.cancer.gov/). The DNA methylation data comprised 430 samples, with 50 normal and 380 cancer tissue samples, while the transcriptome data comprised 424 samples, with 50 normal and 374 cancer tissue samples. The ICGC Data Portal included 86 cancer projects from 17 regions in Asia, Australia, Europe, and North and South America (11). The Liver Cancer-RIKEN, Japan (LIRI-JP) project containing 243 HCC tumor and 202 nontumor samples was downloaded from the ICGC Data Portal (https://dcc.icgc.org). We also downloaded series matrix files of the expression microarray series (GSE36376, GSE14520, GSE54236, GSE64041) from the GEO database (https://www.ncbi.nlm.nih.gov/geo/). The platforms for the four series were GPL10558 (Illumina Human HT-12 V4.0 expression beadchip), GPL571 (Affymetrix Human Genome U133A 2.0 Array), GPL 6480 (Agilent_014850 whole Human Genome Microarray 4X44K G4112F), and GPL6244 (Affymetrix Human Gene 1.0 ST Array). Series matrix files were processed with the Strawberry Perl software (version 5.32.1) to convert the gene probe IDs to gene symbol codes. Because series matrix files had been preliminarily normalized and log base 2 transformed, we ultimately performed data normalization using the normalizeBetweenArrays function of the R Limma package. Then, we used the Cancer Cell Line Encyclopedia (CCLE) dataset to explore *FAM57A* expression in HCC cell lines (https://portals.broadinstitute.org/ccle/). Comparison of FAM57A expression between tumor and nontumor samples was performed using the R Limma package (12).

### Analysis for the Clinical and Prognostic Value of FAM57A Expression

HCC samples with complete clinical information from the TCGA database were selected for the subsequent clinical significance analysis. The clinicopathological characteristics included age, sex, pathological grade, distant metastasis (M), tumor stage (T), clinical stage, lymphatic metastasis (N), survival time, and survival status. We analyzed the association of FAM57A expression with clinicopathological characteristics and their prognostic significance. We also utilized the Kaplan–Meier plotter database to validate the correlation of FAM57A expression with prognosis of HCC patients (https://kmplot.com/analysis/). In addition, we investigated the correlations between FAM57A methylation and expression or prognosis, and further performed joint survival analysis based on a combination of FAM57A methylation and expression.

### Gene Set Enrichment Analysis (GSEA)

To explore the potential cascades in which FAM57A might be involved in HCC, the GSEA software (version 4.1.0) was utilized. In this study, we categorized the samples as high- and low-FAM57A phenotypes based on the median score of the FAM57A level from the TCGA database. The c2.cp.kegg.v7.3.symbols.gmt gene sets were used as the internal gene sets. The phenotypic enrichment pathways were delineated using the normalized enrichment score (NES) and the nominal *P*-value (13). False discovery rate (FDR) q-value <0.05 and nominal *p*-value were considered to be statistically significant.

### Gene Coexpression and GO/KEGG Enrichment Analyses

We screened the coexpression genes of FAM57A expression from the TCGA database. The *p*-value<0.001 and the absolute value of Pearson correlation coefficient |R|≥0.5 were selected as the screening threshold. Then we performed Gene Ontology (GO) and Kyoto Encyclopedia of Genes and Genomes (KEGG) enrichment analyses for the coexpression genes by the R package “clusterProfiler” (version 3.18.1), which used multiple databases, including Disease Ontology database, Network of Cancer Gene database, Gene Ontology database, KEGG database, and Reactome Pathway database (14). Before performing enrichment analysis, gene symbol codes were converted to Entrez ID by using the human genome annotation package “org.Hs.eg.db”. Adjusted *p*-values<0.05 were chosen as the threshold of significance.

### Analysis of Immune Landscape Related to the Expression Level of FAM57A

The Tumor Immune Estimation Resource version 2.0 (TIMER2.0) database (https://timer.cistrome.org/) is commonly utilized to evaluate and visualize the profiles of TIICs among various cancer types. In this study, we analyzed the correlation of FAM57A expression with the abundance of TIICs or gene markers of TIICs in HCC *via* this database. The gene markers of TIICs comprised markers of B cells, T cells (general), CD8+ T cells, tumor-associated macrophages (TAMs), monocytes, neutrophils, natural killer (NK) cells, M1 macrophages, M2 macrophages, dendritic cells (DCs), T-helper 1 (Th1) cells, T-helper 2 (Th2) cells, Tregs cells, follicular helper T (Tfh) cells, and T-helper 17 (Th17) cells. In addition, we evaluated the prognostic potential of FAM57A expressions in HCC based on the immune cell subgroup *via* the Kaplan–Meier plotter database. Immune checkpoint inhibitors have shown therapeutic potential for patients with advanced HCC in clinical trials. Therefore, we further carried out differential and correlation analysis to evaluate the correlation between FAM57A expression and immune checkpoint genes. In the differential analysis, we divided the samples into high- and low-FAM57A expression groups according to the median level of FAM57A expression. For the correlation analysis, our screening criteria were an |R|≥0 and *p*-value<0.05.

### Immunohistochemistry (IHC) Staining

We retrospectively collected 30 pairs of paraffin-embedded HCC and adjacent nontumor tissue samples obtained during operations performed between May 2019 and December 2020 from the Third Hospital of Hebei Medical University. This study was approved by the Ethics Committee of the Third Hospital of Hebei Medical University (approval no. K2020-043-1) and conformed with the principles of the Helsinki Declaration. Informed consent was waived by the Ethics Committee of the Third Hospital of Hebei Medical University given the anonymity of the data and the retrospective nature of our study.

IHC staining of paraffin-embedded sections was carried out manually as reported previously (15). Tissue samples were deparaffinized, hydrated, and blocked with 3% hydrogen peroxide followed by high-pressure antigen retrieval. The samples were then blocked in 10% goat serum and incubated overnight with rabbit antihuman primary FAM57A antibody (1:100, Invitrogen, USA) at 4°C. Next, they were probed with goat antirabbit horseradish peroxidase (HRP) combined with a secondary antibody (ZSGB-Bio, China) at 37°C. Finally, they were incubated with 3,3’-diaminobenzidine (DAB) and stained with hematoxylin. Two experienced pathologists independently assessed the samples. Each tissue sample was scored based on staining intensity and the percentage of tumor cells stained. The scores ranged from 0 to 3: a score of 0–1 was considered negative, and a score of 2–3 was a positive stain.

### Cell Culture and Transfection

Human HCC cell lines (HepG2 and Huh7) were obtained from China Cell Culture Center (Shanghai, China) and maintained under standard conditions. Small interfering RNA (siRNA)-FAM57A and universal negative control (NC) were synthesized by JTS scientific (Wuhan, China). The sequences of FAM57A-siRNA1, FAM57A-siRNA2, and FAM57A-siRNA3 were as follows: (siRNA1) 5’-CCCGGGAAUAUGUGUGGUUTT-3’; (siRNA2) 5’-CCGCCUCAUGAUCACACAUTT-3’; (siRNA3) 5’-CCCUUCUGUACAAGGUGAATT-3’. The negative siRNA was used as the control (NC, 5’-UUCUCCGAACGUGUCACGUTT-3’). siRNA transfections were carried out using Lipofectamine2000 (Invitrogen, USA) according to the manufacturer’s instructions. The transfected cells were used for subsequent experiments as described below.

### Quantitative Real-Time Reverse Transcription Polymerase Chain Reaction (qRT–PCR) and Western Blotting

The qRT-PCR and western blotting assays were carried out to determine knockdown efficiency. A total of 3.5×10^5^ HCC cells were seeded into 6-well plates before transfection. Total RNA was extracted 48 h after transfection using a Trizol reagent (Ambion, USA) following the protocols in the product manual. The obtained RNA was used to synthesize cDNA for PCR using the PrimeScript RTMaster Mix and TB Green Premix Ex Taq II (TaKaRa, Japan). The primers for GAPDH and FAM57A were F: 5’-CCCACTCCTCCACCTTTGAC-3’, R: 5’-CATACCAGGAAATGAGCTTGACAA-3’ and F: 5’-GCTTGCCCGGGAATATGTG-3’, R: 5’-GCTGCTTTAGCTGTGCGAC-3’, respectively. The relative mRNA levels of TLCD3A were determined by the 2–ΔΔCT method.

Total protein was extracted 72 h after transfection by RIPA lysis buffer with proteinase inhibitors (Beyotime, China), and its concentrations were detected with the BCA Protein Assay Kit (MCE, USA). After separation with 10% SDS-PAGE and transferred onto PVDF membranes (Millipore, USA), the proteins on the membranes were blocked with 5% fat-free milk at room temperature for 2 h. Then, the membranes were incubated with β-actin (1:1,000, ZSGB-Bio, China) and FAM57A (1:1,000, Invetrigen, USA) primary antibodies overnight at 4°C, and incubated again with secondary antibodies (1:3,000, Proteintech, China) for 1.5 h at room temperature. Lastly, the protein bands were detected with enhanced chemiluminescent substrate (ECL; ZSGB-Bio, China).

### Cell Proliferation Assay

Cell proliferation was assessed using the Cell Counting Kit-8 (CCK-8) assay kit (MCE, USA). HepG2 and Huh7 cells were seeded into 96-well plates at 5×10^3^ cells per well and grown overnight; then after transfection, the medium was incubated for 24, 48, and 72 h, respectively. Next, CCK-8 solution (10 μl) was added and incubated at 37°C for 2 h. Finally, the optical density (OD) values were recorded using a CMax Plus microplate reader (SpectraMax, China) at 450 nm. Each sample was analyzed in triplicate.

### Flow Cytometry Assay

HepG2 and Huh7 cells were seeded into 6-well plates at 5×10^5^ cells per well. Forty-eight hours after transfection, the cells were collected, washed twice with PBS, and double-stained with an annexin V-fluorescein isothiocyanate and propidium iodide (Annexin V-FITC/PI) solution (Solarbio, China). Cell apoptosis was detected by flow cytometry (Beckman, FC500, USA) according to the manufacturer’s protocol. Each sample was analyzed in triplicate.

### Statistical Analysis

Statistical analyses of IHC and cell experimental data were performed using SPSS (version 23.0) and GraphPad software (version 8.0). The IHC and cell experimental results were carried out with McNemar’s test and one-way ANOVA followed by Dunnett or Bonferroni *post hoc* analysis, respectively. Other statistical analyses from the public databases were performed using R software (version 4.0.3). The median value of FAM57A expression was considered as its cutoff value. The association of clinicopathologic characteristics with FAM57A expression was analyzed by the Wilcoxon rank sum test or Kruskal–Wallis rank sum test and logistic regression. The effect of FAM57A expression on survival was evaluated with the Kaplan–Meier method, followed by the log-rank test. The effects of FAM57A expression and other clinicopathological characteristics on survival were compared with univariate and multivariate Cox regression. Receiver operating characteristic (ROC) curves and the area under the curve (AUC) were used to evaluate the diagnostic and predictive performance of FAM57A. Correlation analysis of gene expression was evaluated with the Spearman correlation coefficient and statistical significance. A p-value or FDR value less than 0.05 was considered statistically significant.

## Results

### FAM57A Expression Was Upregulated in HCC

**Figure 1:**
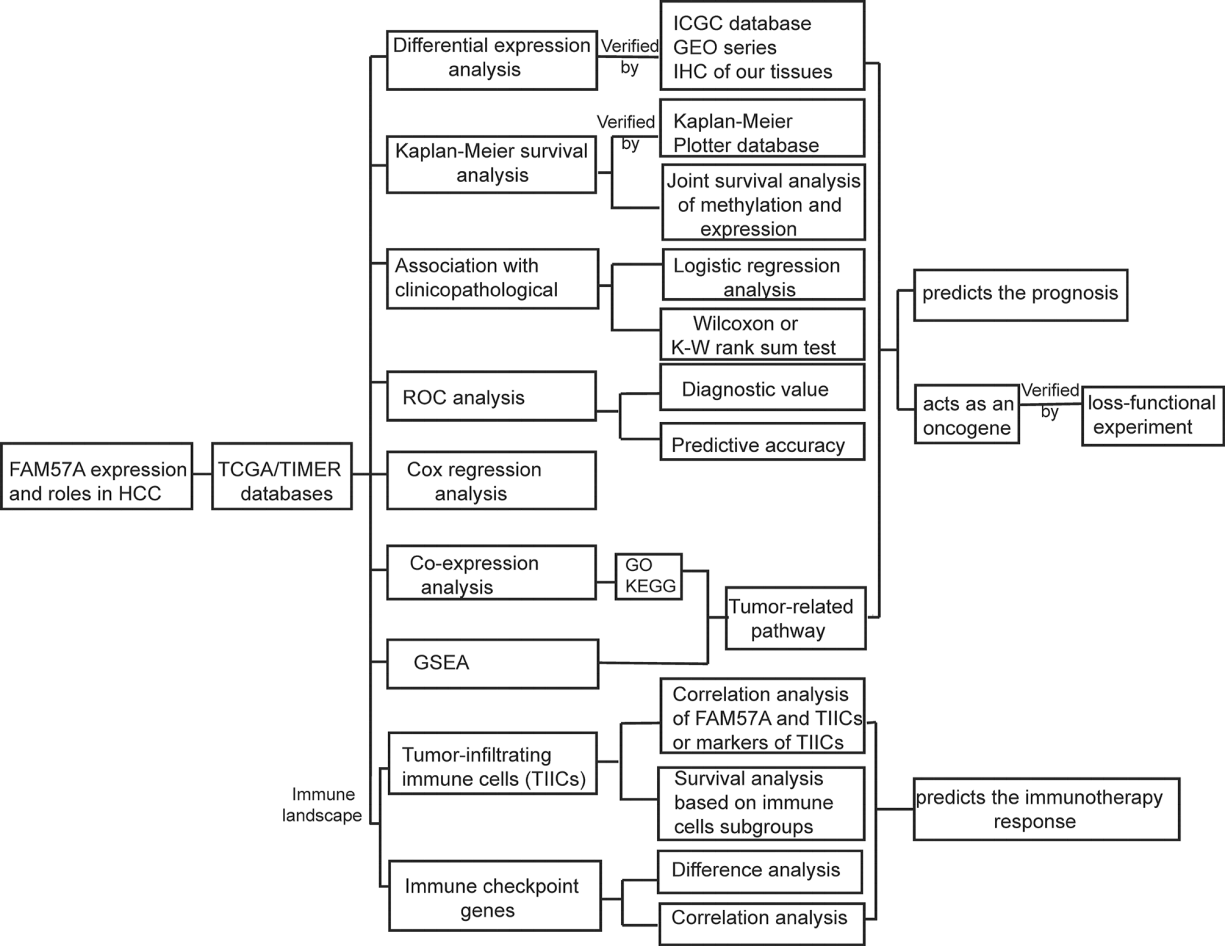
Analysis workflow of this study. GSEA, Gene set enrichment analysis; GO, Gene Ontology; HCC, hepatocellular carcinoma; IHC, immunohistochemistry; KEGG, Kyoto Encyclopedia of Genes and Genomes; ROC, receiver operating characteristic.

**Figure 2:**
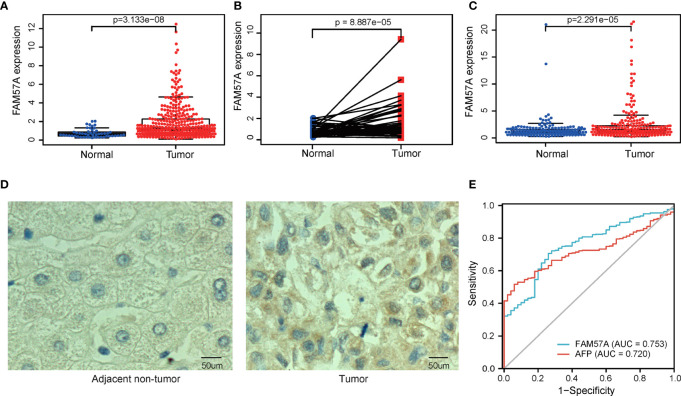
The differentiated expression of FAM57A in samples. **(A)** FAM57A expression in HCC tissues and nontumor tissue from the TCGA database. **(B)** FAM57A level in 50 paired cancerous and noncancerous liver samples from the TCGA database. **(C)** FAM57A level in HCC tissues and nontumor tissue from the ICGC database. **(D)** Representative immunohistochemical images for paired HCC tissues from our hospital (scale bars=50μm). **(E)** AUCs of AFP and FAM57A expression based on the TCGA database. AUC, area under the curve.

The analysis process of the present study is shown in **Figure 1**. We first analyzed the expression of *FAM57A* based on the TCGA database. **Figure 2A** demonstrates that the FAM57A level in the tumor group was markedly higher than that in the nontumor group. To induce individual differences, we compared the expression of FAM57A in 50 paired cancerous and noncancerous liver samples. The result showed that FAM57A was upregulated in cancerous samples (**Figure 2B**). The ICGC and GEO databases showed consistent results (**Figure 2C** and **Supplementary Figure 1**). Moreover, the results of 30 paired HCC/noncancerous IHC stained tissues from our hospital further confirmed the high protein expression level of FAM57A in tumor tissues (76.7% *vs* 46.7%, p<0.05; **Figure 2D**). Taken together, these results provided evidence that FAM57A expression was upregulated in HCC. More intriguingly, the diagnostic performance of FAM57A expression was not inferior to AFP in HCC patients based on the TCGA database (**Figure 2E**).

### FAM57A Expression Negatively Correlated With Overall Survival in HCC

**Figure 3:**
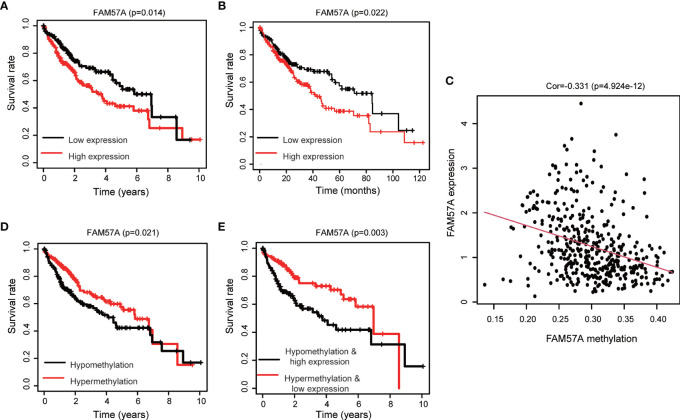
Impact of the FAM57A level and methylation on survival in HCC patients. **(A)** Correlation of OS with FAM57A expression in TCGA. **(B)** Correlation of OS with FAM57A expression in the K-M plotter. **(C)** Negative correlation between FAM57A expression and methylation. **(D)** Correlation of OS with FAM57A methylation in TCGA. **(E)** Joint survival analysis of FAM57A methylation and expression in TCGA. OS, overall survival.

The survival analysis illustrated that the high level of FAM57A was markedly associated with worse OS (**Figure 3A**). Moreover, the result based on the online Kaplan–Meier plotter database confirmed an inverse relationship of FAM57A expression to OS (**Figure 3B**). Additionally, FAM57A expression was negatively related to methylation (**Figure 3C**). This prompted further analysis of the effect of FAM57A methylation on survival, and a joint survival analysis combined methylation levels of FAM57A with the corresponding gene expression levels. Hypomethylation of FAM57A significantly correlated with an unfavorable prognosis, and patients with hypomethylation and overexpression of FAM57A had worse survival relative to those with hypermethylation and lower level expression (**Figures 3D, E**). Therefore, expression levels of *FAM57A* could predict the survival of HCC.

### FAM57A Level Was Linked to the Clinicopathological Characteristics of HCC Patients

**Figure 4:**
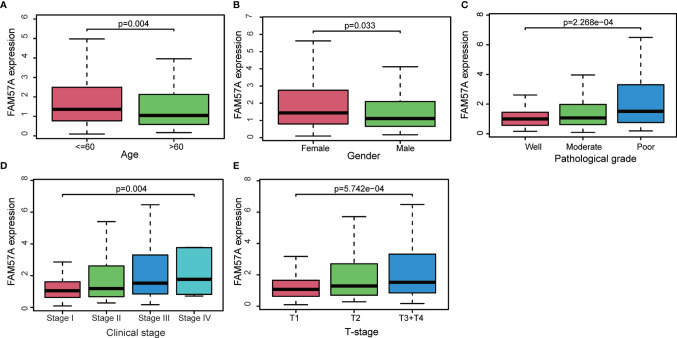
Association with FAM57A expression and clinicopathologic characteristics. **(A)** Age. **(B)** Gender. **(C)** Pathological grade. **(D)** Clinical stage. **(E)** T stage.

FAM57A expression markedly related to age (*p*=0.004), gender (*p*=0.033), pathologic tumor grade (*p*=2.268e-04), clinical stage (*p*=0.004), and T stage (*p*=5.742e-04) based on the TCGA database (**Figure 4**). Univariate logistic regression analysis indicated that upregulated FAM57A expression in HCC was significantly associated with high pathologic grade (OR= 2.26 for poor *vs* well), clinical stage (OR= 2.34 for Stage III *vs* stage I), and T stage (OR=2.09 for T3+T4 *vs* T1) (all *p*-values <0.05; **Table 1**). The interpretation upregulated FAM57A expression correlated with poor clinicopathologic characteristics.

**Table 1:** Association with FAM57A expression and clinicopathologic characteristics (logistic regression).

Clinicopathological characteristics	Total (N)	Odds ratio for FAM57A expression	*p-*value
Age (years)			
>60 *vs* ≤60	370	0.61 (0.40-0.91)	0.017^*^
Gender			
Male *vs* female	371	0.62 (0.40-0.96)	0.033^*^
Pathological grade			
Moderate *vs* well	232	1.07 (0.58-1.99)	0.826
Poor *vs* well	189	2.26 (1.20-4.33)	0.012^*^
Clinical stage			
Stage II *vs* stage I	257	1.34 (0.79-2.26)	0.267
Stage III *vs* stage I	256	2.34 (1.38-4.03)	0.002^*^
Stage IV *vs* stage I	176	2.01 (0.33-15.58)	0.449
Tumor stage (T)			
T2 *vs* T1	275	1.44 (0.87-2.38)	0.155
T3+T4^a^ *vs* T1	274	2.09 (1.26-3.50)	0.005^*^
Lymphatic metastasis			
Positive *vs* negative	256	3.05 (0.38-62.07)	0.337
Distant metastasis			
Positive *vs* negative	270	1.00 (0.12-8.43)	1.000

^a^Since the number of patients with T4 was very small, we pooled patients with T3 and T4 for analysis. ^*^P < 0.05.

### FAM57A Expression Was an Independently Prognostic Predictor in HCC Patients

**Figure 5:**
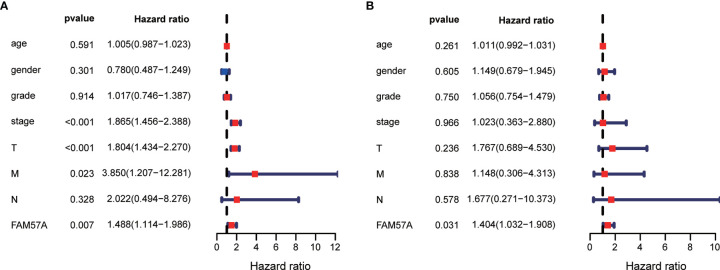
Univariate and multivariate Cox regression analyses of the FAM57A level and other clinicopathological characteristics. **(A)** Univariate Cox regression analysis. **(B)** Multivariate Cox regression analysis.

**Figure 6:**
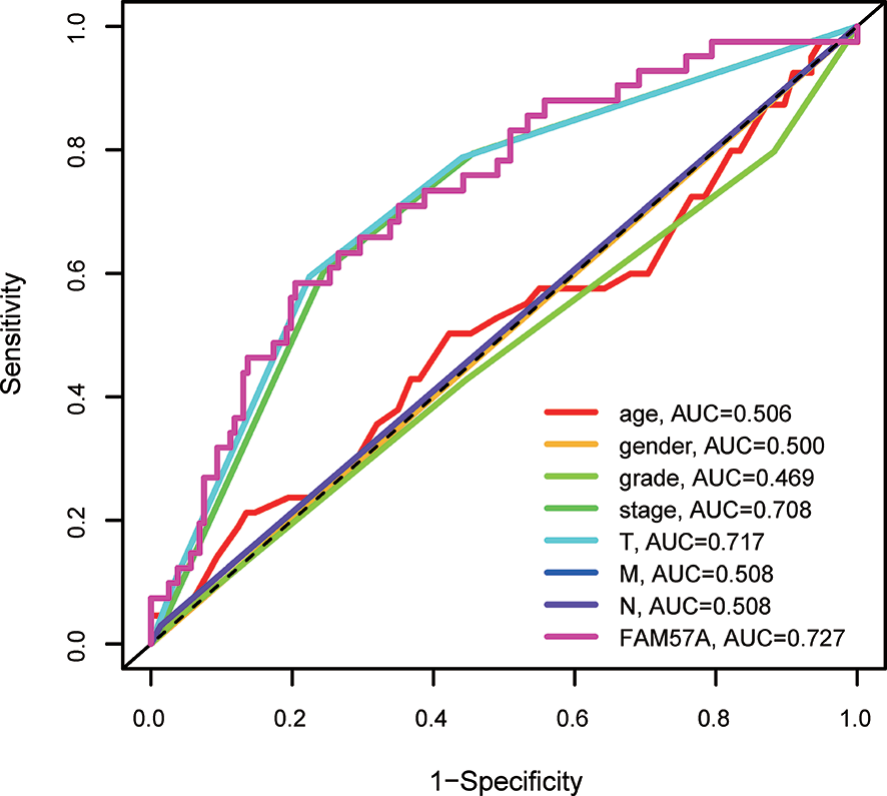
AUCs of prognostic predictors (including grade, gender, age, clinical stage, T stage, M stage, N stage and FAM57A expression). AUC, area under the curve.

Univariate Cox regression analysis demonstrated that elevated FAM57A was significantly linked to poor OS (HR: 1.488, 95% CI: 1.114–1.986, *P*=0.007; **Figure 5A**). Although other clinicopathological characteristics including clinical stage, T stage, and M stage also correlated with OS according to univariate Cox regression analysis, only FAM57A expression retained a significant association with OS by multivariate Cox regression analysis (HR: 1.404, 95% CI: 1.032–1.908, *P*=0.031; **Figure 5B**). Therefore, FAM57A expression was uniquely correlated with the clinical course of HCC patients. To elucidate the predictive accuracy of FAM57A expression, ROC was performed. Compared to other predictive factors (gender, age, pathologic grade, clinical stage, M stage, N stage, and T stage), the AUC of the FAM57A expression was the highest (AUC=0.727, **Figure 6**).

### GSEA

**Figure 7:**
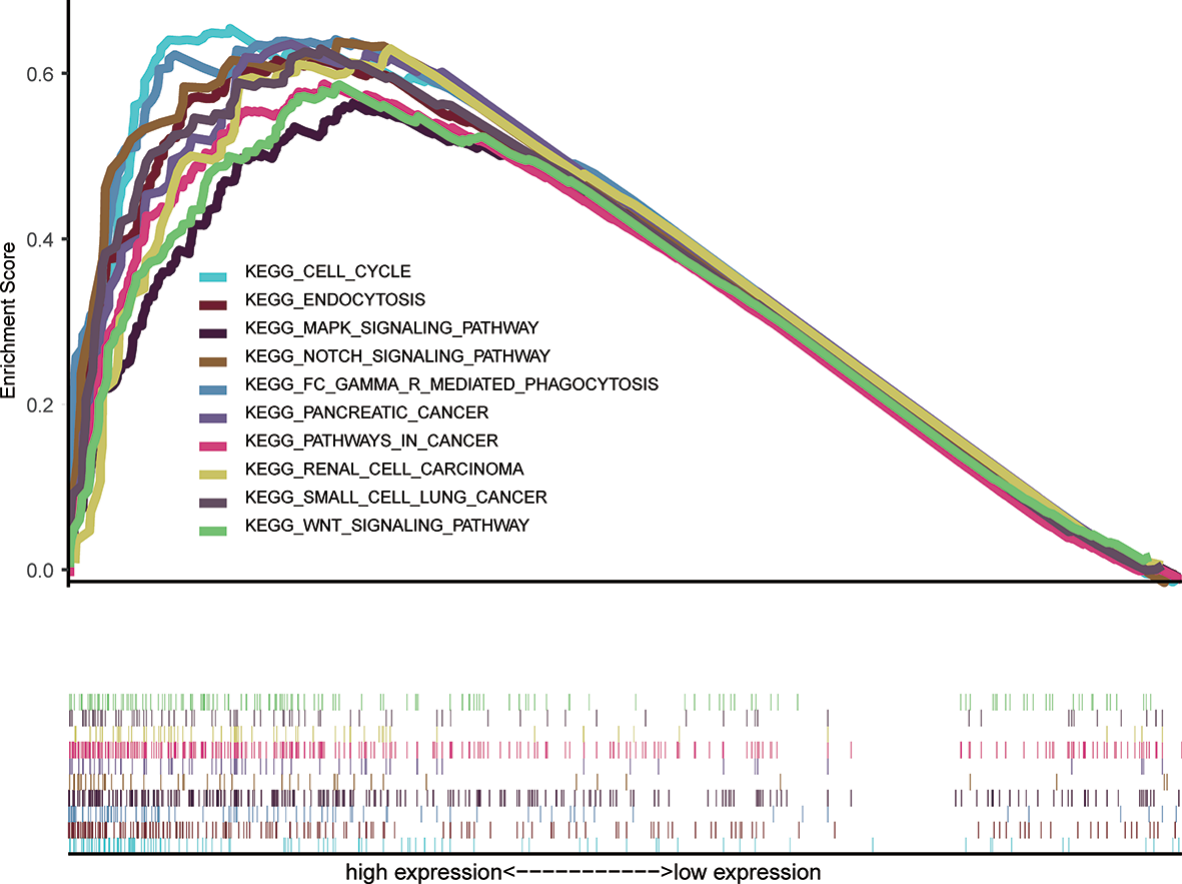
GSEA of the KEGG signaling pathway activated by high expression of FAM57A in HCC. GSEA, Gene set enrichment analysis; KEGG, Kyoto Encyclopedia of Genes and Genomes.

We performed GSEA between high and low FAM57A level phenotypes to explore its related pathways based on the TCGA database. According to NES and FDR values, we selected markedly enriched KEGG pathways. A total of 94 pathways showed substantial enrichment in FAM57A high-level phenotypes (**Supplementary Table 1**). The most typically enriched signaling pathways are displayed in **Figure 7**, indicating that the multiple tumor-related pathways are linked to the FAM57A high-level phenotype.

### Gene Coexpression and GO/KEGG Enrichment Analysis

**Figure 8:**
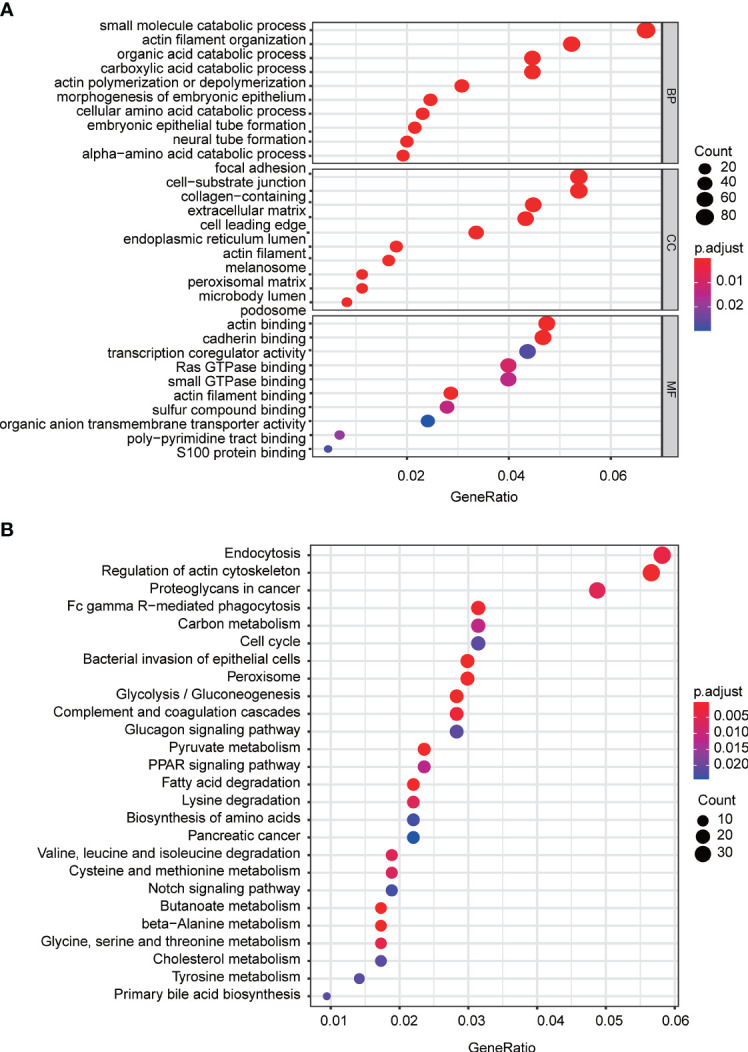
The top 10 of GO terms and KEGG pathways associated with the coexpression genes of FAM57A. **(A)** GO terms (including BP, CC, and MF). **(B)** KEGG pathways. BP, biological process; CC, cellular component; MF, molecular function; GO, Gene Ontology; KEGG, Kyoto Encyclopedia of Genes and Genomes.

To further explore the potential mechanisms mediated by FAM57A in HCC, we conducted coexpression and GO/KEGG enrichment analysis based on the TCGA database. According to |R| and *p*-value, 1,460 genes coexpressed with FAM57A were chosen for subsequent GO/KEGG enrichment analysis. GO annotation revealed that 356 biological processes (BPs), 92 molecular functions (MFs), and 26 cellular component (CC) terms significantly enriched (adjust *p*-value <0.05). The top 10 terms of GO annotation are displayed in **Figure 8A**, suggesting that coexpression genes of FAM57A may exhibit a regulatory effect on HCC *via* focal adhesion, cell-substrate junction, cadherin binding, and small molecule catabolic process. Moreover, 26 KEGG pathways were markedly enriched (adjust *p*-value <0.05, **Figure 8B**). Of note, endocytosis, proteoglycans in cancer, cell cycle, and pancreatic cancer were markedly enriched.

### FAM57A Affected Cell Proliferation and Cell Apoptosis *In Vitro*

**Figure 9:**
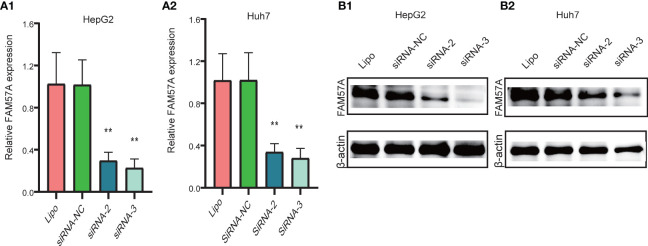
Detection of FAM57A knockdown efficiency. **(A1, A2)** qRT-PCR assay for HepG2 and Huh7 cells, respectively. **(B1, B2)** Western blotting analysis in HepG2 and Huh7 cells, respectively. ^**^p < 0.01 compared to Lipo and siRNA-NC. Lipo, Lipofectamine 2000.

**Figure 10:**
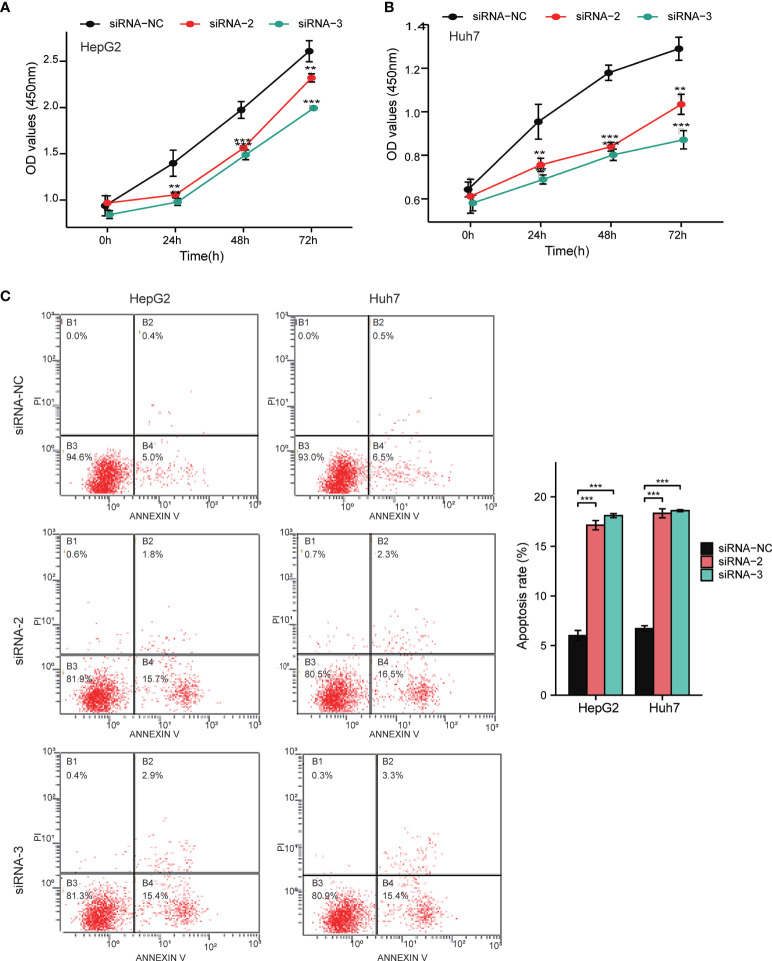
Detection of cell proliferation and cell apoptosis by CCK-8 assay and flow cytometry assay. **(A, B)** Cell proliferation in HepG2 and Huh7 cells determined by CCK-8 assay. **(C)** Cell apoptosis in HepG2 and Huh7 cells determined by flow cytometry assay. Data were shown as mean ± standard deviation of three technical replicates. ^**^p < 0.01, ^***^p < 0.001 compared to siRNA-NC.

Notably, the CCLE database revealed that HCC cell lines expressed relatively high levels of FAM57A in 1,062 cell lines representing 37 distinct cancer types (**Supplementary Figure 2**). Developed from the baseline FAM57A levels in HCC cell lines from the CCLE database, we constructed siRNAs into two HCC cell lines with relatively high expression levels, namely HepG2 and Huh7. Three FAM57A siRNAs were tested for knockdown efficiency. Among them, siRNA-2 and siRNA-3 knocked down FAM57A with greater efficiency based on the results of qRT-PCR and Western blotting (**Figures 9A, B**) and were therefore used in subsequent cell proliferation and cell apoptosis assays. Both siRNA-2 and siRNA-3 significantly inhibited HepG2/Huh7 cell proliferation and induced cell apoptosis compared to controls (**Figure 10**). These results suggested that FAM57A could promote cell proliferation in HCC.

### FAM57A Expression Was Correlated With Immune Infiltration Level in HCC

**Figure 11:**
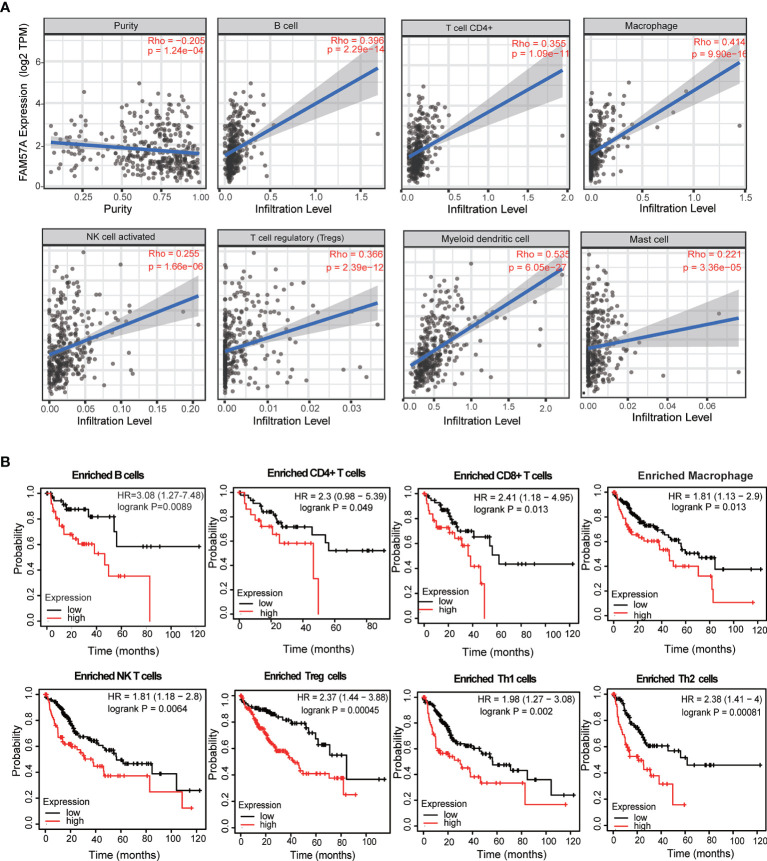
Correlation analysis of the FAM57A level and immune cell infiltration levels and survival analysis based on the immune cell subgroup using the TIMER database and the Kaplan–Meier plotter database, respectively. **(A)** FAM57A expression positively correlates with immune infiltration levels of B cells, CD4+ T cells, macrophage cells, NK cells, Tregs, myeloid dendritic cells, and mast cells. **(B)** High FAM57A level enriched in B cells, CD4+ T cells, CD8+ T cells, macrophages, NK T cells, Treg T cells, Th1 cells, and Th2 cells had worse OS in HCC.

To assess the relationship between FAM57A and immune infiltration in HCC, we used TIMER to analyze the correlation of the FAM57A level with TIICs or gene markers of TIICs. The former showed that FAM57A expression was significantly correlated with B cells (r=0.396, p=2.29e-14), CD4+ T cells (r=0.355, p=1.09e-11), macrophages (r=0.414, p=9.90e-16), NK cells (r=0.255, p=1.66e-06), T regulatory cells (r=0.366, p=2.39e-12), myeloid dendritic cells (r=0.535, p=6.05e-27), and mast cells (r=0.221, p=3.66e-05) in HCC (**Figure 11A**). The latter result indicated that the FAM57A level was positively associated with most gene markers of TIICs (**Table 2**). These findings strongly implicated *FAM57A* as a major regulator of tumor immune infiltration in HCC. Also, upregulated FAM57A expression had worse survival. Therefore, we performed Kaplan–Meier plotter analyses of FAM57A expression based on the immune cell subgroup to assess whether FAM57A might affect the survival partly through immune infiltration. We found that high FAM57A levels in HCC in enriched B cells (p=6.9e-03), CD4+ T cells (p=0.049), CD8+ T cells (p=0.013), macrophages (p=0.013), NK T cells (p=5.4e-03), regulatory T cells (p=4.5e-04), type 1 T helper cells (p=2e-03), and type 2 T helper cells (p=6.1e-04) had a worse prognosis (**Figure 11B**).

**Table 2:** Correlation analysis between FAM57A expression and gene markers of immune cells in TIMER.

Description	Gene markers	Without adjustment		Adjusted by purity	
		Cor	*P*	Cor	*P*
CD8+ T cell	CD8A CA8B	0.276 0.253	** **	0.209 0.184	** **
T cell (general)	CD3D CD3E CD2	0.374 0.345 0.341	** ** **	0.315 0.28 0.276	** ** **
B cell	CD19 CD79A	0.249 0.28	** **	0.181 0.195	** **
Monocyte	CD86 CD115 (CSF1R)	0.433 0.416	** **	0.385 0.362	** **
TAM	CCL2 CD68 IL10	0.375 0.417 0.362	** ** **	0.294 0.357 0.301	** ** **
M1 Macrophage	INOS (NOS2) IRF5 COX2 (PTGS2	0.098 0.349 0.443	NS ** **	0.088 0.357 0.4	NS ** **
M2 Macrophage	CD163 VSIG4 MS4A4A	0.222 0.32 0.278	** ** **	0.147 0.259 0.208	** ** **
Neutrophils	CD66b (CEACAM8) CD11b (ITGAM) CCR7	0.137 0.389 0.309	** ** **	0.132 0.34 0.235	** ** **
Natural killer cell	KIR2DL1 KIR2DL3 KIR2DL4 KIR3DL1 KIR3DL2 KIR3DL3 KIR2DS4	-0.007 0.181 0.11 0.042 0.067 0.032 0.066	NS ** ** NS NS NS NS	-0.051 0.133 0.075 0.008 0.032 0.012 0.072	NS ** NS NS NS NS NS
Dendritic cell	HLA-DPB1 HLA-DQB1 HLA-DRA HLA-DPA1 BDCA-1 (CD1C) BDCA-4 (NRP1) CD11C (ITGAX)	0.368 0.299 0.327 0.343 0.302 0.475 0.427	** ** ** ** ** ** **	0.297 0.227 0.253 0.274 0.23 0.444 0.393	** ** ** ** ** ** **
Th1	T-bet (TBX21) STAT4 STAT1 IFN-γ(IFNG) TNF-α(TNF)	0.153 0.336 0.399 0.185 0.356	** ** ** ** **	0.067 0.293 0.382 0.134 0.309	NS ** ** ** **
Th2	GATA3 STAT6 STAT5A IL13	0.408 0.244 0.436 0.009	** ** ** NS	0.363 0.237 0.395 -0.017	** ** ** NS
Treg	FOXP3 CCR8 STAT5B TGFβ(TGFB1)	0.038 0.359 0.184 0.616	NS ** ** **	0.007 0.32 0.228 0.58	NS ** ** **
Tfh	BCL6 IL21	0.212 0.032	** NS	0.23 0.017	** NS
Th17	STAT3 IL17A	0.313 0.059	** NS	0.282 0.059	** NS

TAM, tumor-associated macrophage; Th, T helper cell; Tfh, Follicular helper T cell; Treg, regulatory T cell; Cor, R value of Spearman’s correlation. **P < 0.05; NS, not significant.

### FAM57A Expression Was Correlated With Immune Checkpoint Genes in HCC

**Figure 12:**
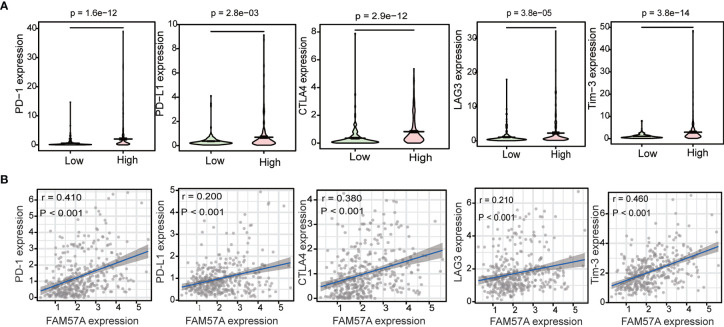
Correlation between FAM57A expression and immune checkpoint genes (PD-1, PD-L1, CTLA-4, LAG3, and Tim-3). **(A)** Differential analysis of immune checkpoint genes with high and low expression levels of FAM57A. **(B)** Correlation analysis between FAM57A expression and immune checkpoint genes.

To investigate the relationship between FAM57A and immune checkpoint genes, we initially compared the expression of immune checkpoint genes, including programmed cell death-1 (PD-1), programmed cell death ligand 1 (PD-L1), cytotoxic T lymphocyte-associated protein 4 (CTLA-4), lymphocyte activation gene 3 (LAG3), and T-cell immunoglobulin mucin 3 (Tim-3) between high- and low-FAM57A expression groups. The expression of immune checkpoint genes was higher in the high-FAM57A group than in the low-FAM57A group (**Figure 12A**). The relationships between FAM57A and immune checkpoint genes was further investigated with correlation analysis confirming that FAM57A expression was positively correlated with immune checkpoint genes (**Figure 12B**). These analyses indicated that high expression levels of FAM57A were directly proportional to PD-1, PD-L1, CTLA-4, LAG3, and Tim-3.

## Discussion

As a novel membrane-associated gene, FAM57A has been reported to exert an oncogenic function in lung cancer and head and neck cancers (7–10). However, prior to this study, the expression of FAM57A and its potential roles in HCC were essentially unknown. Herein, we confirmed that the expression of FAM57A was upregulated in HCC, and this upregulation was linked to advanced stages and worse clinical outcomes of HCC. In addition, functional enrichment analyses further indicated that FAM57A was involved in multiple tumor-related pathways. More importantly, functional assay revealed that FAM57A knockdown significantly inhibited cell proliferation and induced cell apoptosis in HCC cells. Also interconnected, FAM57A expression was positively correlated with TIICs, gene markers of TIICs, as well as immune checkpoint genes.

Bioinformatics analysis and gene expression profiling have been widely used in the study of tumorigenesis mechanisms and the discovery of biomarkers (4, 5). Numerous studies have identified many potential biomarkers by reanalyzing and synthesizing vast amounts of data deposited in public databases (16–18). In the present study, we combined results from such databases with our own data acquired from the IHC of tissue samples to ultimately verify that FAM57A levels were upregulated in HCC. Although the diagnostic power of AFP has been questioned, AFP remains the most effective biomarker for HCC diagnosis at present (19). Our study demonstrated that the expression levels of FAM57A exhibited a relatively satisfactory diagnostic performance in HCC patients compared to AFP. We also found that the upregulation of FAM57A expression predicted a worse prognosis for HCC patients. DNA methylation has been recognized as a key epigenetic regulatory mechanism, crucial to the initiation and progression of tumors. The abnormal genetic expression also plays a significant role in tumorigenesis. Consequently, joint analyses of DNA methylation and gene expression have been utilized to identify the pathogenesis of cancer (20, 21). In this study, DNA methylation and gene expression combined survival analysis showed that *FAM57A* could serve as an indicator for prognosis assessments of HCC patients.

The present study also illustrated that the FAM57A expression level was directly correlated with the clinical stage, pathological grade and T stage, which suggested that HCC patients with high FAM57A expression were more likely to experience progression to advanced stages. The expression levels of FAM57A were also correlated with age and gender but were not significantly correlated with M and N stages. However, due to the small numbers of M1 and N1 patients in the TCGA database, it was not possible to define the precise association of FAM57A expression with lymphatic and distant metastasis. Subsequent studies with larger sample sizes would be necessary to confirm this finding. Further analyses indicated that FAM57A expression could independently predict the prognosis in HCC patients. Moreover, the predictive accuracy of FAM57A expression was slightly better than that of the clinical stage. At present, the clinical stage at diagnosis is regarded as the best indicator for disease prognosis (22). Nevertheless, patients with the same TNM stage might follow a widely variable clinical course with markedly different prognoses due to the inherent molecular diversity of the disease (23). Therefore, a validated, accurate molecular-based approach would be highly valuable to identify patients with poor prognoses and to optimize their individual therapies. Collectively, these results strongly suggested that FAM57A expression might serve as such a prognostic biomarker for HCC.

In order to further explore the potential mechanism mediated by FAM57A in HCC, we performed GSEA, coexpression analysis, and GO and KEGG analyses based on the TCGA database. Both GSEA results and the KEGG pathway enrichment of genes coexpressed with FAM57A indicated that FAM57A was likely involved in multiple tumor-related pathways, such as pancreatic cancer, proteoglycans in cancer, cell cycle, NOTCH, MAPK, and WNT signaling pathways. Certainly, uncontrolled cell proliferation due to a disordered cell cycle is a hallmark of cancer (24). Previously, studies demonstrated that MAPK, WNT, and NOTCH signaling pathways participated in the progression of HCC (25–27). From the results of the sequential bioinformatics analysis and IHC, we concluded that FAM57A might function as an oncogene in the progression of HCC. Therefore, the final step in our overall investigation was to conduct an *in vitro* cell functional assay to preliminarily verify the role of FAM57A in HCC. Based on the baseline FAM57A level in HCC cells from the CCLE database, we constructed siRNAs into two HCC cell lines with relatively high expression levels. Our results of this loss-functional experiment showed that FAM57A knockdown significantly inhibited cell proliferation and induced cell apoptosis in HCC cells. This certainly has future relevance for the development of a targeted inhibitor of FAM57A.

Another major finding of this study was that the expression levels of FAM57A correlated with TIICs and gene markers of TIICs in HCC. Our results revealed the potential regulatory role of FAM57A in tumor immune infiltration. In addition, our results indicated that high FAM57A levels could affect the survival of HCC patients through immune infiltration. A series of immune checkpoint genes, including CTLA-4, PD-1, PD-L1, and LAG-3, have been confirmed to be involved in the induction and maintenance of immune tolerance in HCC (28–30). Moreover, immune checkpoint inhibitors have shown therapeutic potential for patients with advanced HCC in clinical trials (31, 32). Therefore, we further investigated the association between FAM57A expression and immune checkpoint genes and found that high expression levels of FAM57A were directly proportional to PD-1, PD-L1, CTLA-4, LAG-3, and Tim-3. The above analyses suggested that FAM57A could also be used as a biomarker to predict immunotherapy responses for HCC patients.

The principal benefit of this study was the comprehensive analysis of FAM57A expression and its potential roles in HCC derived from validated publicly accessible databases and experimental verification. Moreover, the reliability of FAM57A as a prognostic marker for HCC was characterized through multiple dimensions. Regarding treatment, our study showed that FAM57A could also be used as a biomarker to predict immunotherapy responses for HCC patients. However, our study still had certain limitations. First, we only initially explored the impact of FAM57A on HCC cell proliferation and cell apoptosis. Second, the actual detailed mechanism through which FAM57A influences HCC needs further experimentation.

In summary, we comprehensively analyzed the expression of FAM57A and its potential roles in HCC through bioinformatics analysis and experimental validation. Our study demonstrated that the upregulation of FAM57A expression appears to promote the progression of HCC and could serve as a predictor of poor prognosis and immunotherapy response for HCC patients.

## Data Availability Statement

The original contributions presented in the study are included in the article/**Supplementary Material**. Further inquiries can be directed to the corresponding author.

## Ethics Statement

The studies involving human participants were reviewed and approved by the Ethics Committee of the Third Hospital of Hebei Medical University (approval no. K2020-043-1). Written informed consent for participation was not required for this study in accordance with the national legislation and the institutional requirements.

## Author Contributions

CZ designed and supervised the study. JW and YL performed the study and participated in data analysis. CZ, JW, and YL revised the article that was written. CZ reviewed the manuscript. All authors contributed to the article and approved the submitted version.

## Funding

This work was supported by the Key Research and Development project of Hebei province [grant number 20377768D] and the Science and Technology Research and Development project of Handan city [grant number 19422083010-22].

## Conflict of Interest

The authors declare that the research was conducted in the absence of any commercial or financial relationships that could be construed as a potential conflict of interest.

## Publisher’s Note

All claims expressed in this article are solely those of the authors and do not necessarily represent those of their affiliated organizations, or those of the publisher, the editors and the reviewers. Any product that may be evaluated in this article, or claim that may be made by its manufacturer, is not guaranteed or endorsed by the publisher.
