# Supplementary material for: Integrated Analysis of FAM57A Expression and Its Potential Roles in Hepatocellular Carcinoma
Source: Front Oncol. 2021 Nov 1;11:719973. doi: 10.3389/fonc.2021.719973 (PMC8591096; doi:10.3389/fonc.2021.719973)
Supplement: Supplementary file 1 [file DataSheet_1.zip › Supplementary figure legends.docx]

**Supplementary Figure 1** The differentiated expression of FAM57A in GEO series. **(A)** GSE36376 **(B)** GSE14520 **(C)** GSE54236 **(D)** GSE64041

**Supplementary Figure 2** The expression levels of FAM57A in 1062 cell lines representing 37 distinct cancer types based on the CCLE database.
