# Supplementary material for: Integrated Analysis of FAM57A Expression and Its Potential Roles in Hepatocellular Carcinoma
Source: Front Oncol. 2021 Nov 1;11:719973. doi: 10.3389/fonc.2021.719973 (PMC8591096; doi:10.3389/fonc.2021.719973)
Supplement: Supplementary file 1 [file DataSheet_1.zip › Supplementary Table 1.docx]

Supplementary Table 1 Gene sets enriched in phenotype high

| Gene set name | NES | NOM  p-val | FDR  q-val |
| --- | --- | --- | --- |
| KEGG_Pathogenic_escherichia_coli_infection | 2.126432 | 0 | 0 |
| KEGG_Pathways_in_cancer | 2.074617 | 0 | 0.0052 |
| KEGG_Endocytosis | 2.073624 | 0 | 0.003467 |
| KEGG_Vasopressin_regulated_water_reabsorption | 2.062851 | 0 | 0.0026 |
| KEGG_MAPK_signaling_pathway | 2.057458 | 0 | 0.00208 |
| KEGG_Oocyte_meiosis | 2.055582 | 0 | 0.001733 |
| KEGG_Gnrh_signaling_pathway | 2.047722 | 0 | 0.001755 |
| KEGG_Regulation_of_actin_cytoskeleton | 2.028867 | 0 | 0.003333 |
| KEGG_Purine_metabolism | 2.019614 | 0 | 0.003581 |
| KEGG_Small_cell_lung_cancer | 2.007866 | 0 | 0.003541 |
| KEGG_Axon_guidance | 2.006749 | 0 | 0.003301 |
| KEGG_Gap_junction | 2.000895 | 0 | 0.003436 |
| KEGG_NOD_like_receptor_signaling_pathway | 1.980094 | 0 | 0.004149 |
| KEGG_WNT_signaling_pathway | 1.978913 | 0 | 0.003921 |
| KEGG_Renal_cell_carcinoma | 1.978131 | 0 | 0.003968 |
| KEGG_Cytosolic_dna_sensing_pathway | 1.972967 | 0 | 0.00406 |
| KEGG_Epithelial_cell_signaling_in_helicobacter_pylori_infection | 1.963626 | 0 | 0.004165 |
| KEGG_Fc_gamma_r_mediated_phagocytosis | 1.961592 | 0 | 0.003992 |
| KEGG_Neurotrophin_signaling_pathway | 1.961121 | 0 | 0.003782 |
| KEGG_Toll_like_receptor_signaling_pathway | 1.959966 | 0 | 0.003683 |
| KEGG_Apoptosis | 1.958833 | 0 | 0.003562 |
| KEGG_P53_signaling_pathway | 1.954756 | 0 | 0.003626 |
| KEGG_Pancreatic_cancer | 1.940807 | 0 | 0.004532 |
| KEGG_Phosphatidylinositol_signaling_system | 1.940595 | 0 | 0.004343 |
| KEGG_Snare_interactions_in_vesicular_transport | 1.937985 | 0 | 0.004169 |
| KEGG_Cell_cycle | 1.930037 | 0 | 0.004512 |
| KEGG_Long_term_depression | 1.92233 | 0 | 0.00461 |
| KEGG_Homologous_recombination | 1.922198 | 0 | 0.004446 |
| KEGG_TGF_beta_signaling_pathway | 1.92138 | 0 | 0.004325 |
| KEGG_Bladder_cancer | 1.921353 | 0 | 0.004181 |
| KEGG_Rig_i_like_receptor_signaling_pathway | 1.919641 | 0.004158 | 0.004215 |
| KEGG_NOTCH_signaling_pathway | 1.91609 | 0 | 0.004233 |
| KEGG_Melanogenesis | 1.897876 | 0 | 0.005739 |
| KEGG_Glycosphingolipid_biosynthesis_lacto_and_neolacto_series | 1.897736 | 0 | 0.00557 |
| KEGG_Focal_adhesion | 1.897281 | 0.002053 | 0.005494 |
| KEGG_FC_epsilon_ri_signaling_pathway | 1.893395 | 0 | 0.005809 |
| KEGG_Dorso_ventral_axis_formation | 1.889026 | 0 | 0.005892 |
| KEGG_Progesterone_mediated_oocyte_maturation | 1.887712 | 0 | 0.00579 |
| KEGG_VEGF_signaling_pathway | 1.883051 | 0 | 0.005897 |
| KEGG_Vibrio_cholerae_infection | 1.880372 | 0.004184 | 0.00596 |
| KEGG_Hypertrophic_cardiomyopathy_hcm | 1.875935 | 0.004057 | 0.00608 |
| KEGG_Leishmania_infection | 1.875056 | 0.005917 | 0.005958 |
| KEGG_MTOR_signaling_pathway | 1.874647 | 0 | 0.00586 |
| KEGG_Chemokine_signaling_pathway | 1.874185 | 0.002083 | 0.005807 |
| KEGG_RNA_degradation | 1.873455 | 0 | 0.005736 |
| KEGG_Acute_myeloid_leukemia | 1.87194 | 0 | 0.005632 |
| KEGG_Ubiquitin_mediated_proteolysis | 1.871789 | 0 | 0.005538 |
| KEGG_Chronic_myeloid_leukemia | 1.870698 | 0 | 0.005501 |
| KEGG_T_cell_receptor_signaling_pathway | 1.867932 | 0.002114 | 0.005675 |
| KEGG_Long_term_potentiation | 1.865471 | 0 | 0.005727 |
| KEGG_Vascular_smooth_muscle_contraction | 1.864719 | 0 | 0.00565 |
| KEGG_Spliceosome | 1.858009 | 0 | 0.006116 |
| KEGG_ECM_receptor_interaction | 1.857315 | 0.003861 | 0.006089 |
| KEGG_Inositol_phosphate_metabolism | 1.856045 | 0.002066 | 0.006104 |
| KEGG_Pyrimidine_metabolism | 1.851696 | 0.004073 | 0.006245 |
| KEGG_Cytokine_cytokine_receptor_interaction | 1.849222 | 0.006036 | 0.006374 |
| KEGG_Tight_junction | 1.840269 | 0.00432 | 0.007027 |
| KEGG_Dilated_cardiomyopathy | 1.836881 | 0.003992 | 0.007165 |
| KEGG_Leukocyte_transendothelial_migration | 1.836293 | 0.006237 | 0.007208 |
| KEGG_Lysosome | 1.832827 | 0.006135 | 0.007498 |
| KEGG_JAK_STAT_signaling_pathway | 1.832714 | 0.002075 | 0.007375 |
| KEGG_Arrhythmogenic_right_ventricular_cardiomyopathy_arvc | 1.82773 | 0.003937 | 0.007613 |
| KEGG_Prostate_cancer | 1.820365 | 0 | 0.008389 |
| KEGG_B_cell_receptor_signaling_pathway | 1.818534 | 0.004115 | 0.008392 |
| KEGG_ERBB_signaling_pathway | 1.817711 | 0 | 0.008298 |
| KEGG_Glioma | 1.805246 | 0.002075 | 0.009841 |
| KEGG_Ether_lipid_metabolism | 1.782486 | 0 | 0.011888 |
| KEGG_Melanoma | 1.761763 | 0 | 0.013898 |
| KEGG_Adherens_junction | 1.761568 | 0 | 0.013697 |
| KEGG_Natural_killer_cell_mediated_cytotoxicity | 1.749861 | 0.011905 | 0.015138 |
| KEGG_Base_excision_repair | 1.743228 | 0.006148 | 0.01618 |
| KEGG_Regulation_of_autophagy | 1.741572 | 0.005929 | 0.016253 |
| KEGG_Glycosaminoglycan_biosynthesis_chondroitin_sulfate | 1.740898 | 0.003976 | 0.016175 |
| KEGG_Non_small_cell_lung_cancer | 1.736039 | 0.006024 | 0.016727 |
| KEGG_Glycosaminoglycan_biosynthesis_heparan_sulfate | 1.733843 | 0.006 | 0.016911 |
| KEGG_Nucleotide_excision_repair | 1.728391 | 0.007921 | 0.017486 |
| KEGG_Glycerophospholipid_metabolism | 1.720282 | 0.005803 | 0.018583 |
| KEGG_Colorectal_cancer | 1.712473 | 0.004098 | 0.019843 |
| KEGG_DNA_replication | 1.710592 | 0.026804 | 0.019927 |
| KEGG_Amyotrophic_lateral_sclerosis_als | 1.709792 | 0 | 0.019804 |
| KEGG_Insulin_signaling_pathway | 1.704499 | 0.014257 | 0.020486 |
| KEGG_Hedgehog_signaling_pathway | 1.692397 | 0.006329 | 0.022704 |
| KEGG_Calcium_signaling_pathway | 1.669557 | 0.008214 | 0.027063 |
| KEGG_Mismatch_repair | 1.667149 | 0.014344 | 0.027304 |
| KEGG_Basal_cell_carcinoma | 1.653513 | 0.00823 | 0.030005 |
| KEGG_Basal_transcription_factors | 1.652132 | 0.016227 | 0.029888 |
| KEGG_Hematopoietic_cell_lineage | 1.652091 | 0.028056 | 0.029566 |
| KEGG_Type_ii_diabetes_mellitus | 1.647636 | 0.006224 | 0.030742 |
| KEGG_Riboflavin_metabolism | 1.631426 | 0.020367 | 0.034433 |
| KEGG_Viral_myocarditis | 1.630701 | 0.036364 | 0.034263 |
| KEGG_Endometrial_cancer | 1.629523 | 0.022358 | 0.034226 |
| KEGG_Cell_adhesion_molecules_cams | 1.608676 | 0.037267 | 0.039974 |
| KEGG_Prion_diseases | 1.592178 | 0.035785 | 0.044532 |
| KEGG_RNA_polymerase | 1.580333 | 0.035417 | 0.0484 |
