# Supplementary figures and images for: Integrated Analysis of FAM57A Expression and Its Potential Roles in Hepatocellular Carcinoma
Source: Front Oncol. 2021 Nov 1;11:719973. doi: 10.3389/fonc.2021.719973 (PMC8591096; doi:10.3389/fonc.2021.719973)

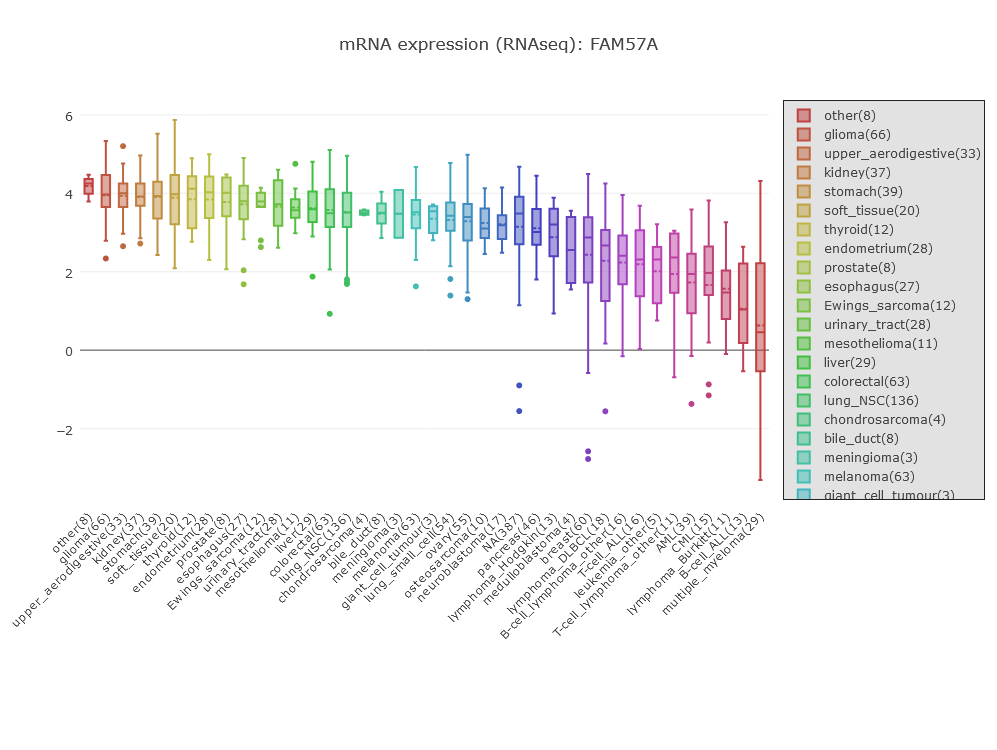

Supplement: Supplementary file 1 [file DataSheet_1.zip › Supplementary Figure 2.tif]

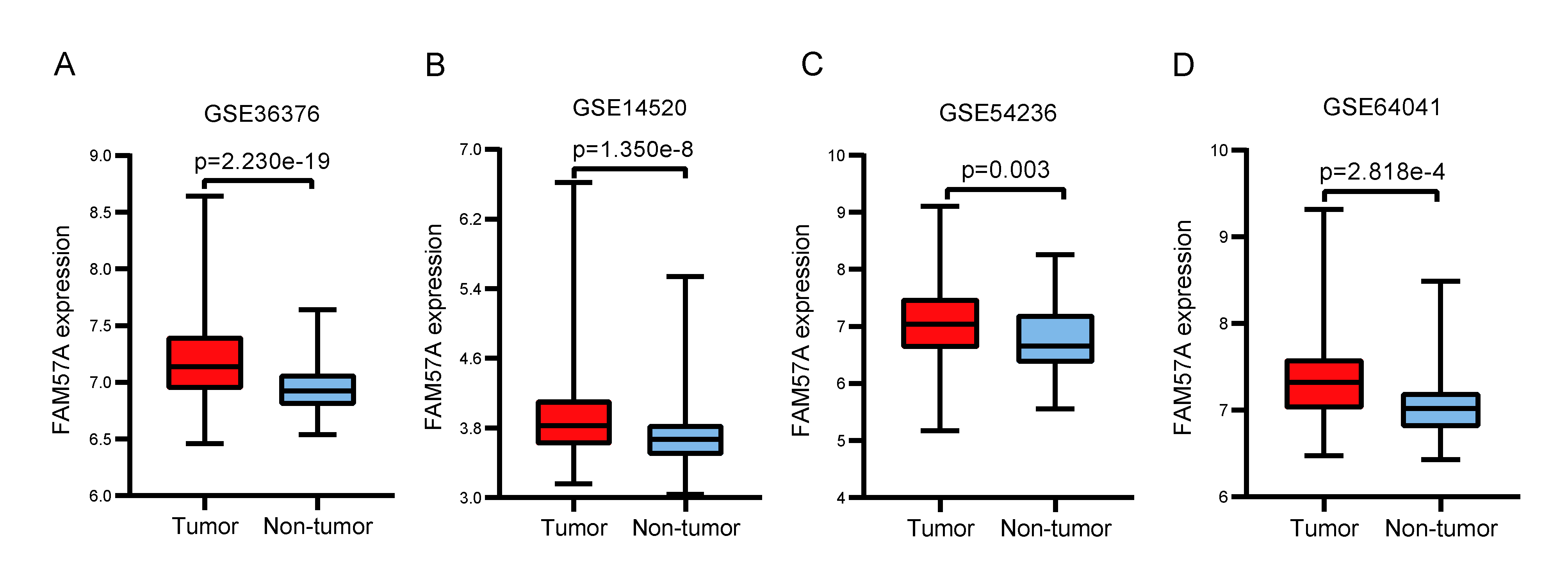

Supplement: Supplementary file 1 [file DataSheet_1.zip › Supplementary Figure 1.tif]
